# Magnetic patterning of Co/Ni layered systems by plasma oxidation

**DOI:** 10.1038/s41598-022-26604-1

**Published:** 2022-12-21

**Authors:** Błażej Anastaziak, Weronika Andrzejewska, Marek Schmidt, Michał Matczak, Ivan Soldatov, Rudolf Schäfer, Mikołaj Lewandowski, Feliks Stobiecki, Christian Janzen, Arno Ehresmann, Piotr Kuświk

**Affiliations:** 1 Institute of Molecular Physics, Polish Academy of Sciences, Smoluchowskiego 17, Poznań, Poland; 2 NanoBioMedical Centre, Adam Mickiewicz University, Wszechnicy Piastowskiej 3, Poznań, Poland; 3 Faculty of Physics, University of Białystok, Białystok, Poland; 4 Leibniz Institute for Solid State and Materials Research (IFW), Helmholtzstraße 20, Dresden, Germany; 5 Institute of Physics and Center for Interdisciplinary Nanostructure Science and Technology (CINSaT), University of Kassel, Kassel, Germany

**Keywords:** Ferromagnetism, Magnetic properties and materials, Surfaces, interfaces and thin films, Magneto-optics, X-rays, Atomic force microscopy, Surface patterning

## Abstract

We studied the structural, chemical, and magnetic properties of Ti/Au/Co/Ni layered systems subjected to plasma oxidation. The process results in the formation of NiO at the expense of metallic Ni, as clearly evidenced by X-ray photoelectron spectroscopy, while not affecting the surface roughness and grain size of the Co/Ni bilayers. Since the decrease of the thickness of the Ni layer and the formation of NiO increase the perpendicular magnetic anisotropy, oxidation may be locally applied for magnetic patterning. Using this approach, we created 2D heterostructures characterized by different combinations of magnetic properties in areas modified by plasma oxidation and in the regions protected from oxidation. As plasma oxidation is an easy to use, low cost, and commonly utilized technique in industrial applications, it may constitute an improvement over other magnetic patterning methods.

## Introduction

Magnetically patterned magnetic materials are promising media for various applications e.g., as high-density storage devices^1–4^, for spin wave propagation in magnonics^5,6^, and in lab-on-a-chip platforms^7–9^. During the last few decades, several techniques have been developed to locally modify the magnetic properties. An especially important requirement in modern methods is the ability to flexibly tailor the magnetic anisotropy without modifying the surface roughness. Such methods include: (1) bombardment with ions, where the magnetic properties are modified by atomic displacements at interfaces as well as by alloying or by supplying energy from ion–solid interactions with least possible sputtering^10–20^ (2) thermal patterning with a hot tip of a scanning probe microscope to perform local field cooling of a ferromagnetic (FM) layer coupled with an antiferromagnetic (AF) layer^21^; (3) different types of oxidation, which may support perpendicular magnetic anisotropy (PMA), either through hybridization between the magnetic transition metal and oxygen atoms^22,23^ or by exchange bias (EB) coupling between AF oxides and the FM^24–26^. Additionally, oxidation allows tailoring the value and the sign of Dzyaloshinskii–Moriya interaction (DMI)^27,28^. The local DMI modification offers new insights regarding the magnetic properties and may improve the performance of magnetic thin films in spintronic and magnonic devices^29,30^. Therefore, it is important to develop a method for local oxidation, in which the magnetic properties can be modified without introducing strong surface damage.

The magnetic patterning method needs to be established for a layered system that meets the material requirements for the applications mentioned above. A Co/Ni bilayer can be such material because it is easy to control the magnetic anisotropy by changing the layers’ thicknesses and, thus, induce PMA. Such systems exhibit also high thermal stability^31^, moderately-high magnetization^32^, low Gilbert damping^33,34^, and high spin polarization^32^. Moreover, for this bilayer, it was found that chemisorption of oxygen on Ni gives the ability to tune the value and sign of DMI^35^. All this indicates that Co/Ni offers a wide spectrum of properties important for applications in spintronics and magnonics.

In that regard, the current work will extend the functionality of Co/Ni systems, as we will show that the magnetic properties can be easily tailored by plasma oxidation (PO) of the top Ni layer. The magnetic post-oxidation state can be controlled by the oxidation time within appropriate choices of thicknesses of the Co and Ni sublayers. The anisotropy modification mechanism includes the reduction of the Ni thickness and the formation of an antiferromagnetic NiO layer. This layer induces an EB interaction supporting additional contribution to PMA^36^.

Here, we provide direct evidence for the reduction of the thickness of metallic Ni through the formation of a surface NiO layer upon PO. This process offers a wide range of possibilities to control the magnetic properties of the Co/Ni system (anisotropy, coercive field, exchange bias). Using this method locally, we created two-dimensional artificial magnetic structures with tunable magnetic properties of protected and oxidized areas.

## Results and discussion

### Changes in structure and composition caused by plasma oxidation

**Figure 1:**
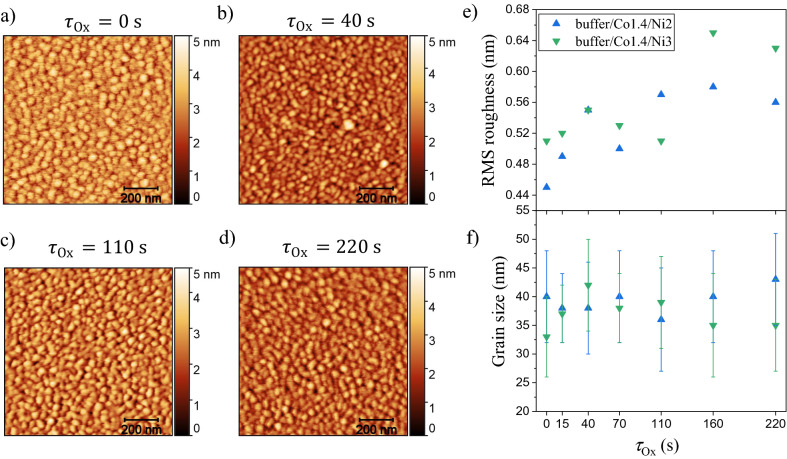
AFM results obtained for the buffer/Co 1.4 nm/Ni 2 nm and buffer/Co 1.4 nm/Ni 3 nm systems: (**a**–**d**) surface topography of buffer/Co 1.4 nm/Ni 2 nm for different oxidation times *τ*_Ox_ = 0 (as deposited state), 40, 110 and 220 s, respectively; (**e**) roughness of layers and (**f**) grain sizes as a function of *τ*_Ox_.

For many applications, the surface needs to be smooth after patterning. To verify how the surface changes after PO, atomic force microscopy (AFM) measurements were performed for two different samples: Co 1.4 nm/Ni 2 nm and Co 1.4 nm/Ni 3 nm. Panels a–d in Fig. 1 show the surface topography in the as-deposited state and after oxidation with different oxidation time (*τ*_Ox_) for the Co 1.4 nm/Ni 2 nm system. In the as-deposited state, the root mean square (RMS) roughness for Co 1.4 nm/Ni 2 nm and Co 1.4 nm/Ni 3 nm are 0.5 and 0.45 nm, respectively. In both systems, the grain size is in the range of ~ 30–50 nm. These results are in good agreement with the data obtained for the Co/Au/Co/Au layered system deposited on a Ti 4 nm/Au 60 nm buffer, where the RMS roughness was 0.65 nm, with a grain size of 40 nm^37^. For thick buffer layers (as compared to the thickness of the other layers), the buffer will mainly determine the grain size and roughness. Taking into account that this type of buffer deposited by magnetron sputtering is polycrystalline with a strong (111) texture^37^, we may assume that Co/Ni system is also polycrystalline with well-visible crystal grains. A comparison of all images recorded before and after oxidation reveals no significant changes in the topography of the samples. This can also be seen in Fig. 1e and f, where only a slight increase of the roughness was found, while the median of the grain size is almost unchanged. This shows that PO does not influence the surface roughness, which remains very smooth.

**Figure 2:**
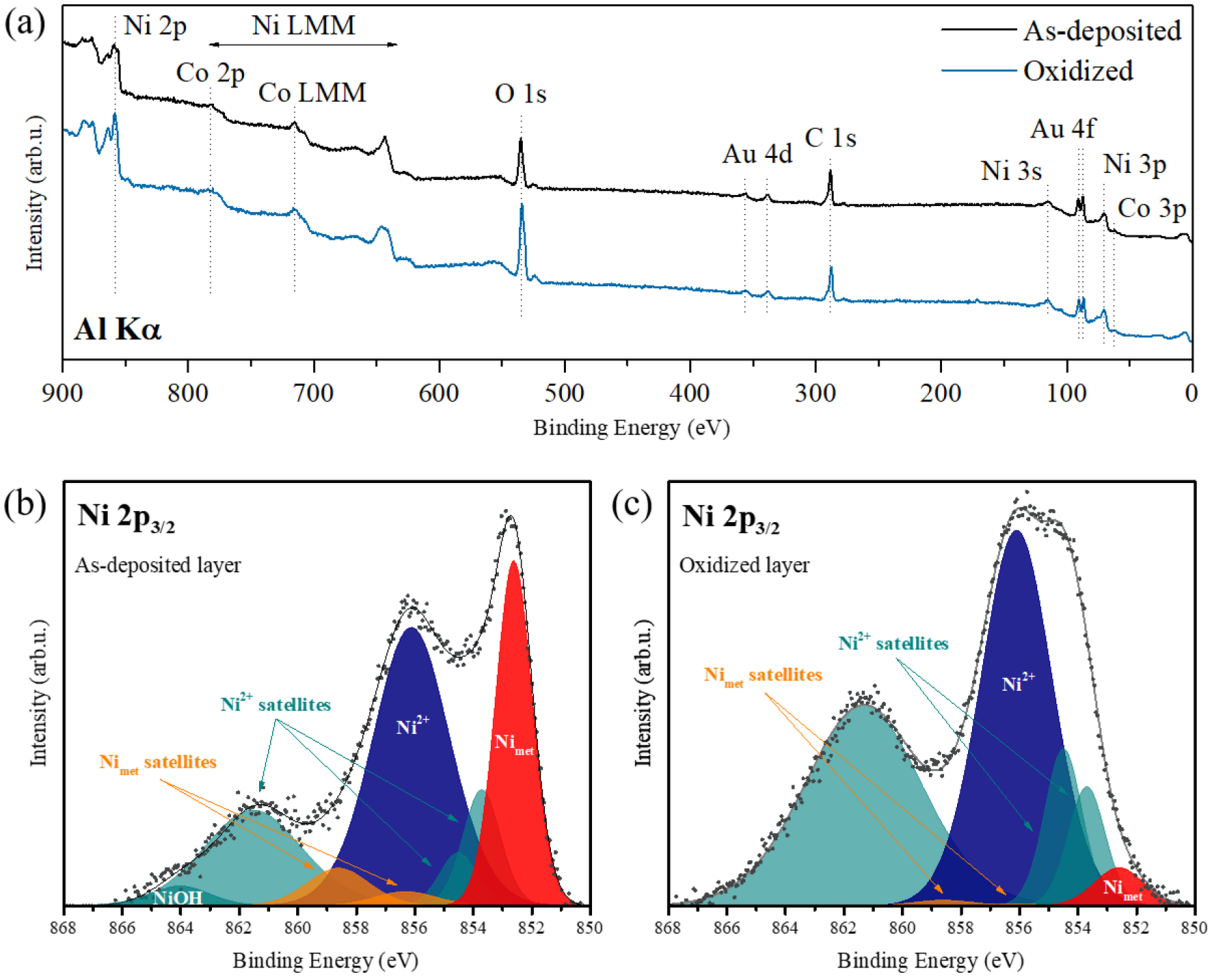
XPS survey (**a**) and detailed Ni 2p_3/2_; (**b**,**c**) spectra recorded for the as-deposited Ti 4 nm/Au 60 nm/Co 1 nm/Ni 2 nm layer and the layer subjected to plasma oxidation *τ*_Ox_ = 220 s. The fitted components in (**b**) and (**c**) correspond to metallic Ni (red), the associated satellite peaks (orange), Ni2+ (blue) and the corresponding satellites (light green), as well as NiOH (dark green).

Next, X-ray photoelectron spectroscopy (XPS) studies were performed to determine the changes in the chemical structure of the layers occurring after PO. XPS is a powerful technique that allows determining of valence states of elements^38^. The survey spectra of the as-deposited sample and the one subjected to PO are shown in Fig. 2a. Both spectra reveal the expected presence of Au, Co, and Ni, as well as traces of C and O. Notably, the intensity of the oxygen signal was much higher for the PO sample. The cobalt signal was weak, which was related to the fact that the element was buried below a nickel/nickel-oxide layer and the kinetic energy of Co 2p photoelectrons is relatively low (leading to a limited escape depth). Moreover, the signal was overlapping with LMM Auger peaks of Ni, which made its identification difficult. The situation was different in the case of gold: even though the layer was buried below the nickel/nickel-oxide and cobalt layers, the high kinetic energy of Au 4f photoelectrons allowed their escape from the sample and detection of a relatively intense signal. Thanks to this, the Au 4f_7/2_ peak could be used for spectra calibration. The AFM studies (Fig. 1) have shown that the sample surface layer possesses a grain structure, so the underling Au buffer layer was expected to have a similar morphology^37^. In such a case, it should be characterized by the Au 4f_7/2_ binding energy (BE) value typical for Au nanoparticles (i.e. 84.3 eV^39,40^). In fact, only with such an assumption the calibrated spectra were exhibiting proper BE values for peaks characteristic of air-exposed samples (such as C 1s or O 1s).

The difference between the chemical states of nickel in the as-deposited layer and the PO-treated structure was determined by analyzing the Ni 2p_3/2_ signals. The results are presented in Fig. 2b and c, respectively. Both lines exhibit complex shapes, indicating the presence of at least two different valence states. The spectrum obtained for the as-deposited layer, shown in Fig. 2b, could be well-fitted using eight spectral components corresponding to metallic Ni (the dominating component centered at 852.6 eV and the characteristic satellite peaks at 856.3 and 858.6 eV), nickel in the Ni^2+^ oxidation state (peaks positioned at 853.7, 854.5, 856.1, 861.3 eV) and the chemisorbed OH groups (864.0 eV). The formation of a surface NiO layer and the presence of OH groups in the case of the as-deposited sample was expected due to exposure to air^41–43^. The positions of the components and the full width at half maximum (FWHM) values used for the fittings, listed in Table 1, were taken from Refs.^44,45^. Most peaks were fixed exactly at the literature positions and with the given FWHM value (except for the peaks positioned at 852.6, 856.1, 861.3 and 864.0 eV, the width of which was allowed to change). Moreover, following Ref.^44^, the intensity ratio relation between the 852.6, 856.3 and 858.6 eV components was set constant. As application of this procedure resulted in a residual standard deviation (STD) value of ~ 1.5—expected based on the linear fitting of the background—the presence of nickel in the Ni^3+^ state was not further considered. The proportion between the Ni_met_ (852.6 eV) and Ni^2+^ (856.1 eV) was approx. 36%:64%, confirming the presence of a surface NiO layer^46,47^ on top of metallic nickel.

**Table 1:** Binding energy (BE) and full width at half maximum (FWHM) values of different Ni_met_ and Ni^2+^ components used for the fittings of the Ni 2p_3/2_ spectra obtained for the as-deposited Ti 4 nm/Au 60 nm/Co 1 nm/Ni 2 nm layer and the layer subjected to plasma oxidation.

	As-deposited sample		Oxidized sample	
	BE (eV)	FWHM	BE (eV)	FWHM
**Main Ni**_**met**_** peak**	852.6	1.47	852.6	1.99
Ni^2+^ satellite (NiO)^45^	853.7	1.50	853.7	1.50
Ni^2+^ satellite (NiO)^38^	854.5	1.50	854.5	1.50
Ni^2+^ satellite (NiO)^45^			855.4	1.50
**Main Ni**^**2+**^** peak**	856.1	3.22	856.1	2.88
Shake-up Ni_met_ surface satellite^45^	856.3	2.50	856.3	2.50
Shake-up Ni_met_ charge-transfer satellite^45^	858.6	2.50	858.6	2.50
Ni^2+^ charge-transfer satellite^48^	861.3	3.24	861.3	4.74
Ni(OH)_2_ peak^49^	864.0	2.37		

Following PO, significant changes in the oxidation state of Ni were observed, as can be seen from the shape of the Ni 2p_3/2_ line presented in Fig. 2c. The spectrum could be still fitted using the same components as in the case of the as-deposited layer (except for the peak related to OH groups), however, the proportion between the Ni_met_ and Ni^2+^ dramatically changed (6.6%:93.4%). This confirmed the formation of a thicker NiO layer at the expense of metallic Ni. Again, the obtained STD value indicates no evident presence of nickel in the Ni^3+^ oxidation state (although it cannot be fully excluded, as the peaks characteristic of Ni^3+^ overlap with those of Ni_met_ and Ni^2+^^50,51^).

To conclude, the as-deposited structure was found to mostly host metallic Ni and some amount of Ni^2+^. This can be rationalized by the formation of a surface NiO layer on top of metallic nickel upon exposure to air^45^. In contrast, the sample subjected to PO was characterized by a dominant contribution from the Ni^2+^ and some from Ni_met_, confirming the formation of a thicker NiO layer at the expense of metallic Ni.

### Modification of magnetic properties caused by plasma oxidation

**Figure 3:**
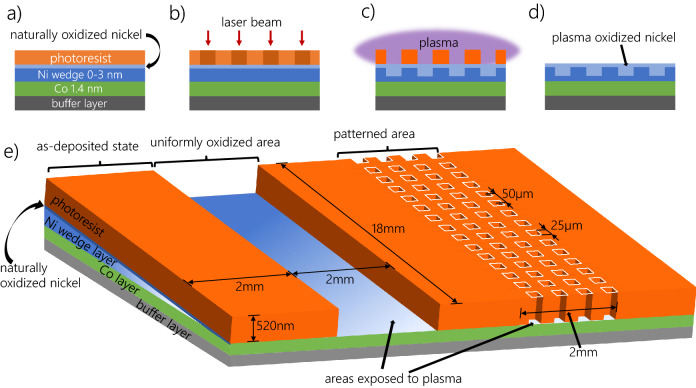
Scheme of magnetic patterning process of Co/Ni system (**a**) that incorporates photolithography (**b**) and PO treatment (**c**). Final state of the sample is shown on (**d**). In panel (**e**) the design of the mask with all important information is presented.

Local tailoring of the magnetic properties was performed for the Co 1.4 nm/Ni-*t*_Ni_ (*t*_Ni_ denotes nickel thickness) wedge (0–3 nm) sample with *τ*_Ox_ = 110 s to show the ability to create 2D heterostructures where the magnetic properties in areas modified by PO are different from those of areas protected from oxidation (matrix) (Fig. 3). For this type of modification, we used a photoresist mask. To carry out this process, it is necessary to select the appropriate resist, which will withstand plasma oxidation. The data sheet for our resist (AP-3510) indicates that it is plasma etching resistant, with etching rates for pure oxygen equal to 165 nm/min^52^. Note that in our experiments we are using a mixture of nitrogen and oxygen (with oxygen content: ~ 36%). Therefore, we expected that this rate will be around three times smaller. Indeed, in our case, the etching rate equals 54 nm/min (see Fig. S1 in the supplementary materials), which allows for a wide time window for local PO through a photoresist mask. To be sure that the resist completely protects chosen areas of the Co/Ni sample from oxidation, we spin-coated a 520 nm thick layer of a photoresist (Fig. 3a). Using direct writing photolithography, we fabricated two distinct areas on top of Co/Ni system (Fig. 3b), which later underwent plasma oxidation (Fig. 3c). The first one consists of periodic arrays of squares with 25 μm side, with the centers of neighboring squares separated by 50 μm. In the second structure, the resist was completely removed, leaving the layer without any pattern serving as a reference for homogeneous plasma oxidation. Both areas span the full length of the sample (18 mm) and have widths of 2 mm (Fig. 3e), which makes them large enough for hysteresis loops measurements using a 0.3 mm laser spot diameter in our polar magneto-optical Kerr effect (P-MOKE) magnetometer. The P-MOKE hysteresis loops were measured as a function of the Ni thickness (wedge-shaped sublayers) for both regions (uniformly oxidized area and patterned region with local oxidation) and also for protected areas (Fig. 3). All these measurements were performed after the resist was removed (Fig. 3d).

**Figure 4:**
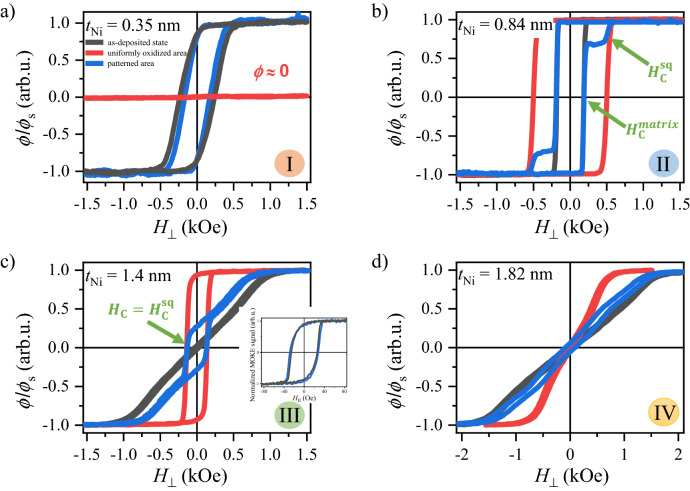
Representative normalized *ϕ*/*ϕ*_s_ P-MOKE hysteresis loops for the buffer/Co 1.4 nm/Ni-*t*_Ni_ (wedge 0–3 nm) system (**a**) *t*_Ni_ = 0.35, (**b**) *t*_Ni_ = 0.84, (**c**) *t*_Ni_ = 1.4, (**d**) t_Ni_ = 1.82 nm. Colors of hysteresis denote the states of the sample: black (matrix)—sample in the as-deposited state; red (uniformly oxidized area)—sample after plasma oxidation with *τ*_Ox_ = 110 s; blue (patterned area)—matrix is protected by the photoresist maintaining its original state, and squares are exposure to plasma. Inset in (**c**) shows the hysteresis loops determined from Kerr microscopy images with in-plane sensitivity for *t*_Ni_ = 1.4 nm for as-deposited and protected areas (matrix). The numbers in the circles correspond to specific *t*_Ni_ ranges in Fig. 5.

**Figure 5:**
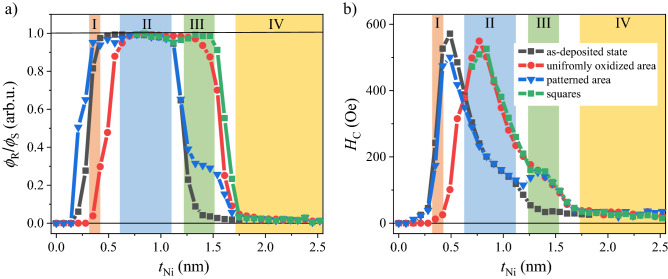
Changes of magnetic properties determined from P-MOKE hysteresis loops registered for three different areas: sample before PO, after uniform PO, and local PO: (**a**) the ratio of remanence to saturation (*ϕ*_R_/*ϕ*_S_) of P-MOKE signal as a function of Ni thickness (*t*_Ni_). Numbers of particular *t*_Ni_ regions corresponds to different magnetic states of the matrix and the squares, respectively: (I) PMA and almost non-ferromagnetic state; (II) PMA and PMA, both with significantly different coercivity; (III) IMA (in-plane magnetic anisotropy) and PMA; (IV) IMA and IMA with different saturation fields; (**b**) coercivity field (*H*_C_) versus thickness of Ni sublayer (*t*_Ni_). Additionally, *H*_C_ and *ϕ*_R_/*ϕ*_S_ extracted for squares from P-MOKE hysteresis loops measured in patterned area are plotted in the regions II-IV (green squares).

We now discuss the behavior of the basic hysteresis loop parameters (remanence to saturation *ϕ*_R_/*ϕ*_S_ of P-MOKE signals and coercivity *H*_C_) measured for the buffer/Co1.4 nm/Ni-*t*_Ni_ (wedge 0–3 nm) layered film in three different areas: (1) not subjected to plasma modification, (2) uniform oxidation and (3) local oxidation (Figs. 4 and 5).

#### As-deposited Co-1.4 nm/Ni-*t*_Ni_ bilayer

First, we discuss the results for the Co/Ni system not subjected to PO, i.e., in an as-deposited state. It displays three distinct ranges of *t*_Ni_ with different magnetic properties of the Co/Ni bilayer (Fig. 5a), which are in good agreement with the data presented in^36^. For the smallest Ni thicknesses (*t*_Ni_ < 0.25 nm) *ϕ* ≈ 0 and the system shows non-ferromagnetic behavior (not shown in Fig. 4). As the Ni thickness increases, a PMA appears up to *t*_Ni_ = 1.2 nm (*ϕ*_R_*/ϕ*_S_ = 1, Fig. 5a), above which a spin reorientation transition occurs (the anisotropy change from PMA to in-plane magnetic anisotropy (IMA)) (0 ≲ *ϕ*_R_*/ϕ* ≲ 1, Fig. 5a). For larger *t*_Ni_, the magnetization lies in the sample plane (*ϕ*_R_*/ϕ*_S_ ≈ 0, Fig. 5a).

#### Co-1.4 nm/Ni-*t*_Ni_ bilayer after uniform plasma oxidation

All *t*_Ni_ regions described for the as-deposited bilayer are also present after uniform oxidation for *τ*_Ox_ = 110 s; however, they appear at larger thicknesses of the Ni layers (Fig. 5a), as was expected based on our recent results^36^. As we have shown, describing the XPS data, the amount of Ni^2+^ is enhanced on the expense of Ni, confirming that PO reduces the effective thickness of the ferromagnetic Ni layer. We earlier found that Ni does not oxidize more than ~ 2 nm even for long oxidation times (up to 220 s). This is also confirmed by XPS measurements as for *t*_Ni_ = 2 nm we still see a weak metallic contribution from Ni after oxidation with 220 s. Since PMA increases for the entire range of oxidation times (up to 220 s), the thickness reduction of the Ni layer cannot be the only source of induced PMA. It is known that the AF layer^24–26,53,54^ supports PMA through the EB coupling between the FM and AF layers. Therefore, the formation of NiO, clearly detected with XPS, brings an additional surface contribution to the PMA, which was postulated in our recent results. The changes in anisotropy are also accompanied by a shift in the maximum *H*_C_ value with the Ni thickness change from *t*_Ni_ = 0.5 to 0.77 nm (Fig. 5b).

#### Co-1.4 nm/Ni-t_Ni_ bilayer after local plasma oxidation

By comparing three states (protected areas, uniform oxidation, and patterned areas), one can see (Fig. 4) that the hysteresis loop for the patterned area is almost the area weighed superposition of the loops for matrix (as-deposited) and the uniformly oxidized area, where the first contribution is three times larger (the surface of the matrix is three times larger than the combined area of squares). This is clearly visible in Fig. 4b where matrix and squares show PMA. In this case, *H*_C_ (the value of the magnetic field at which P-MOKE signal equals zero) of the patterned area corresponds to that of the matrix (*H*_C_ = *H*_C_^matrix^). However, above *t*_Ni_ = 1.25 nm the spin reorientation transition takes place, and the P-MOKE signal at low magnetic fields increase linearly with the magnetic field (the same as in the case of uniformly oxidized area). As a consequence, *H*_C_ for the patterned area reflects the changes of coercive fields of the squares *H*_C_ = *H*_C_^sq^ (Fig. 4c).

To check this interpretation, we plotted *H*_C_ and *ϕ*_R_/*ϕ*_S_ of the squares embedded in the matrix. Both parameters can be determined directly from the hysteresis loops measured for the patterned area in the *t*_Ni_ range (region II in Fig. 5a,b) corresponding to PMA in the matrix and the squares (see Fig. 4b). For higher *t*_Ni_ (region III and IV in Fig. 5a,b), the P-MOKE signal from the squares need to be extracted. In these regions the matrix is characterized by IMA, therefore the P-MOKE signal corresponding to the magnetization reversal of the matrix can be subtracted from hysteresis loops of the patterned area allowing to obtain hysteresis loops for squares only (see Fig. S2). In this way in the regions II–IV values of *H*_C_ and *ϕ*_R_/*ϕ*_S_ corresponding to the squares were determined (green squares in Fig. 5a,b).

The obtained data showed perfect agreement between the magnetic properties after uniform oxidation and local oxidation. This means that the resist protects the sample well against oxidation and only the unprotected part of the sample changes properties in the same way as the uniform oxidation area does. This shows the ability to independently control the magnetic properties of squares (properties depend on oxidation time and *t*_Ni_) and the matrix (properties of the sample before oxidation tuned by *t*_Ni_). Note that the properties of squares and matrix can also be set by the thickness of Co^36^, which offers even more tailoring opportunities.

Therefore, the local PO is a good technique to fabricate such patterned periodic square lattices. Here, we concentrate on obtaining 2D patterns with four different types of structures, so that the magnetic state of squares and matrix can be separately controlled (see Fig. 5a,b):

(I) squares with a non-magnetic state embedded in matrix with PMA,
(II) squares and matrix with PMA with significantly different coercivities,
(III) squares with PMA embedded in matrix with IMA,
(IV) both squares and matrix have IMA with different saturation fields.

**Figure 6:**
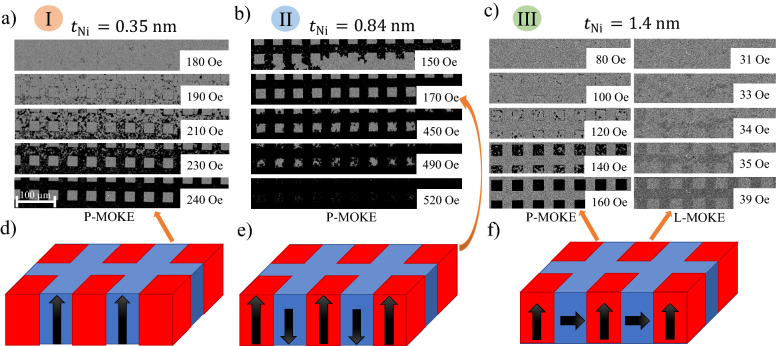
Magnetic patterning by plasma oxidation of buffer/Co 1.4 nm/Ni-*t*_Ni_ (wedge 0–5 nm) system: (**a**–**c**) evolution of magnetic domain structure of matrix and squares after plasma oxidation with *τ*_Ox_ = 110 s, for *t*_Ni_ = 0.35, 0.84 and 1.4 nm, respectively. (**c**) P-MOKE images of magnetic domains for squares and L-MOKE (in-plane sensitivity) for matrix. Numbers describe different magnetic states of the matrix and the modified areas, according to Fig. 5. Schematic picture of magnetic states for matrix (blue) and squares (red), respectively: (**d**) non-magnetic state and PMA; (**e**) PMA and PMA; (**f**) IMA and PMA. Orange arrows indicate images to which the diagrams are related.

The magnetization reversal processes for states I–III registered by Kerr microscopy with perpendicular and in-plane sensitivity are presented in Fig. 6. For state I, the image analysis reveals that the reversal takes place only for a matrix with PMA, as the squares show almost no magneto-optical response (Fig. 6a,d), because the PO destroys the ferromagnetic properties of the Co/Ni system. At small thicknesses, almost the entire Co and Ni layers are oxidized, because the Ni is too thin to protect the Co layer from complete oxidation. For larger thicknesses (0.6 < *t*_Ni_ < 1.1 nm), PO enhances the PMA^36^, which results in a higher coercive field in the squares than in the matrix (*H*_C_^sq^ > *H*_C_^matrix^); therefore, their magnetization switching appears in a higher magnetic field (Fig. 6b). This allows to create artificial magnetic domains (Fig. 6e) at a magnetic field range *H*_C_^matrix^ ≤ *H* ≤ *H*_C_^sq^. Note that this range can be wider for longer oxidation times, because the contribution to the PMA increases with oxidation time^36^.

Above *t*_Ni_ = 1.25 nm, the PO induces PMA. Therefore, Kerr microscopy images with perpendicular sensitivity show strong out-of-plane contrast indicating that the magnetization is oriented perpendicularly to the sample plane, while the magnetization in the matrix lies in the sample plane (note that our investigation is performed on a wedged sample, therefore, it is difficult to distinguish between easy-plane and in-plane anisotropy). To confirm the in-plane magnetization of the matrix, we measured both the domain structure (Fig. 6c) and the hysteresis loop (inset to Fig. 4c) by Kerr microscopy with in-plane sensitivity. Indeed, the magnetic domains in the matrix are well visible (inset to Fig. 6c, right column).

All these measurements reveal that the magnetic properties of squares can be modified to be very different from those of the matrix. Therefore, PO seems very useful for applications that require strong contrast in magnetic properties of locally modified and protected areas. For example, the proposed magnetic patterning technique can be used to fabricate magnonic crystals. Since periodic changes in magnetic properties allow controlling spin wave excitations^55^, and the Co/Ni system has low damping^33,34^, this type of modification looks promising to tune the magnonic band structure^56,57^. The advantage of the proposed technique is that it allows obtaining flat and continuous magnetic materials with laterally modified magnetic properties.

A patterned magnetic system for magnetic beads transportation could be another application for which our patterning method is suitable. Such magnetic patterns should consist of areas with different coercive fields. After reversal of the areas with lower coercivity, domains are formed with strong gradient of magnetic stray fields in the vicinity of domain walls. A proper combination of this stray field with an externally applied magnetic field allows for controlled the transport of magnetic particles, as reported in^8,9,58^, where ratchet-like and topologically protected transport of colloidal particles have been achieved over light-ion bombardment modified magnetic layer systems. In^59^, for example, the coercive field was set to ~ 300 Oe and 80 Oe for the two different regions. Since the longevity of the structure (and therefore its usability) is determined by the values of *H*_C_ in the modified and protected areas, the patterned Co/Ni system modified by PO constitutes a desirable alternative, especially because the coercivity fields can be tuned within relatively large ranges. Considering that PO is an easy to use, low cost, and commonly used technique in industrial applications, it may constitute an improvement over other magnetic patterning methods. It should be emphasized, that PO is performed not only for magnetic tailoring^60^, but it is also widely used to fabricate different non-magnetic structures for example cutting of a single-walled carbon nanotubes to produces carboxylic acid terminated electrodes with separation smaller than 10 nm^61^ or realizing self-aligned gold disks in graphene antidots in which the gap between edges of the gold disks and graphene antidots is ~ 100 nm^62^. All these results show that it is possible to control PO process with high precision using different lithographic masks. This opens a way to carry out magnetic patterning with PO not only in micrometer scale but also in nanoscale range using ultrahigh-resolution electron-beam lithography.

## Conclusions

We have shown that plasma oxidation is a great tool to tune the magnetic properties of Co/Ni bilayers through the reduction of Ni thickness and formation of a NiO layer. Importantly, it does not significantly affect the surface roughness and the grain size. Since the oxidation can be performed locally through the photoresist mask, this process can be used to fabricate 2D structures with different combinations of magnetic properties in the areas modified by plasma oxidation and in the areas protected from oxidation. Because plasma oxidation can be performed on a large scale using commercially available tools, it might serve as an efficient approach for magnetic patterning.

## Material and methods

We investigated different types of buffer/Co-*t*_Co_/Ni-*t*_Ni_ layered films (*t*_Co_ = 1 or 1.4 nm; *t*_Ni_ = 2 or 3 nm), uniform samples and samples with a Ni wedge (0 ≤ *t*_Ni_ ≤ 3 nm, uniformly increasing along 18 mm), patterned with PO. The layered films were deposited onto a naturally oxidized Si(100) wafer covered with a Ti 4 nm/Au 60 nm buffer layer. The deposition chamber was evacuated to a base pressure of *p* = 4.1 × 10^−8^ mbar. All layers (Ti, Au, Co, and Ni) were deposited by magnetron sputtering in an argon atmosphere with *p*_Ar_ = 1.5 × 10^−5^ mbar. After deposition, the samples were oxidized using a Zepto plasma cleaner by Diener Electronic (Germany), following the procedure described in Ref.^36^.

Direct writing photolithography was used to prepare a mask consisting of a 2D periodic pattern of 25 µm × 25 µm squares with periodicities of 50 µm in both perpendicular lateral directions and a reference region (without resist). Selected samples were covered with a positive resist AR-3510 (AllResist GmbH) which in the lithography process were exposed to laser light with a wavelength of 405 nm and a spot diameter of 1 μm.

The hysteresis loops were measured by a P-MOKE magnetometry with a laser beam of 635 nm wavelength focused on a spot of approximately 0.3 mm diameter. The wedge-shaped sample was measured at three different areas: as-deposited state (covered by resist during oxidation), uniformly oxidized area (reference), and patterned area (local oxidation within the squares).

The domain structure was observed using a magneto-optical Kerr microscope^63,64^ with a CMOS camera, from Evico Magnetics GmbH Dresden (Germany). The images were recorded using configurations with perpendicular and in-plane sensitivity. Magnetic domains at field *H *were obtained by subtracting reference images recorded at a saturating magnetic field *H*_ref_ = 7 kOe from the images obtained at *H*. The hysteresis loops for magnetic field applied in the sample plane were determined by plotting the Kerr signal with in-plane intensity from a series of Kerr microscope images recorded during the field changes corresponding to the full magnetization reversal process^65^.

The surface topography was measured by an AFM in tapping mode (*FlexAFM* by Nanosurf, Switzerland) using a tip with typical curvature radius of about 25–30 nm. The images were taken at a scan size of 1 × 1 μm^2^ (256 pixels × 256 pixels). Image analysis were performed by using the Gwyddion 2.6 software^66^. The roughness obtained from the AFM images was expressed as RMS roughness, using an extracting profile along arbitrary lines in two directions. The grain size was determined as the median of measurements for one hundred grains in two perpendicular directions.

XPS studies were performed in an ultra-high vacuum (UHV) chamber (base pressure: 5 × 10^−10^ mbar) from Omicron, Germany. The measurements were carried out for Co 1 nm/Ni 2 nm samples—as-deposited and subjected to PO for 220 s. The spectra were recorded using a non-monochromatic Al K_α_ X-ray source (1486.6 eV) and a channeltron-based hemispherical electron energy analyzer, at a grazing emission and using pass energy values of 50 eV (survey spectra) and 20 eV (regions). All the spectra were calibrated with respect to the position of the Au 4f_7/2_ peak (for details, see the Results and Discussion section) and analyzed using the CasaXPS computer software (Casa Software Ltd). The selection of gold as a reference was related to its high chemical stability and low susceptibility to oxidation. For the fittings of the Ni 2p_3/2_ signals, a Shirley background and a weighted (30%) Gaussian–Lorentzian line shape were used.

All the measurements were performed at room temperature.

## Supplementary Information

Supplementary Figures.

## Data Availability

The data of this study are available from the corresponding authors on reasonable request.
