# Supplementary material for: Magnetic patterning of Co/Ni layered systems by plasma oxidation
Source: Sci Rep. 2022 Dec 21;12:22060. doi: 10.1038/s41598-022-26604-1 (PMC9772314; doi:10.1038/s41598-022-26604-1)
Supplement: Supplementary file 1 — Supplementary Figures. [file 41598_2022_26604_MOESM1_ESM.docx]

Figure S1. Resist thickness reduction with error bars as a function of oxidation time (τ_Ox_). The etch rate was estimated at 54nm/min.


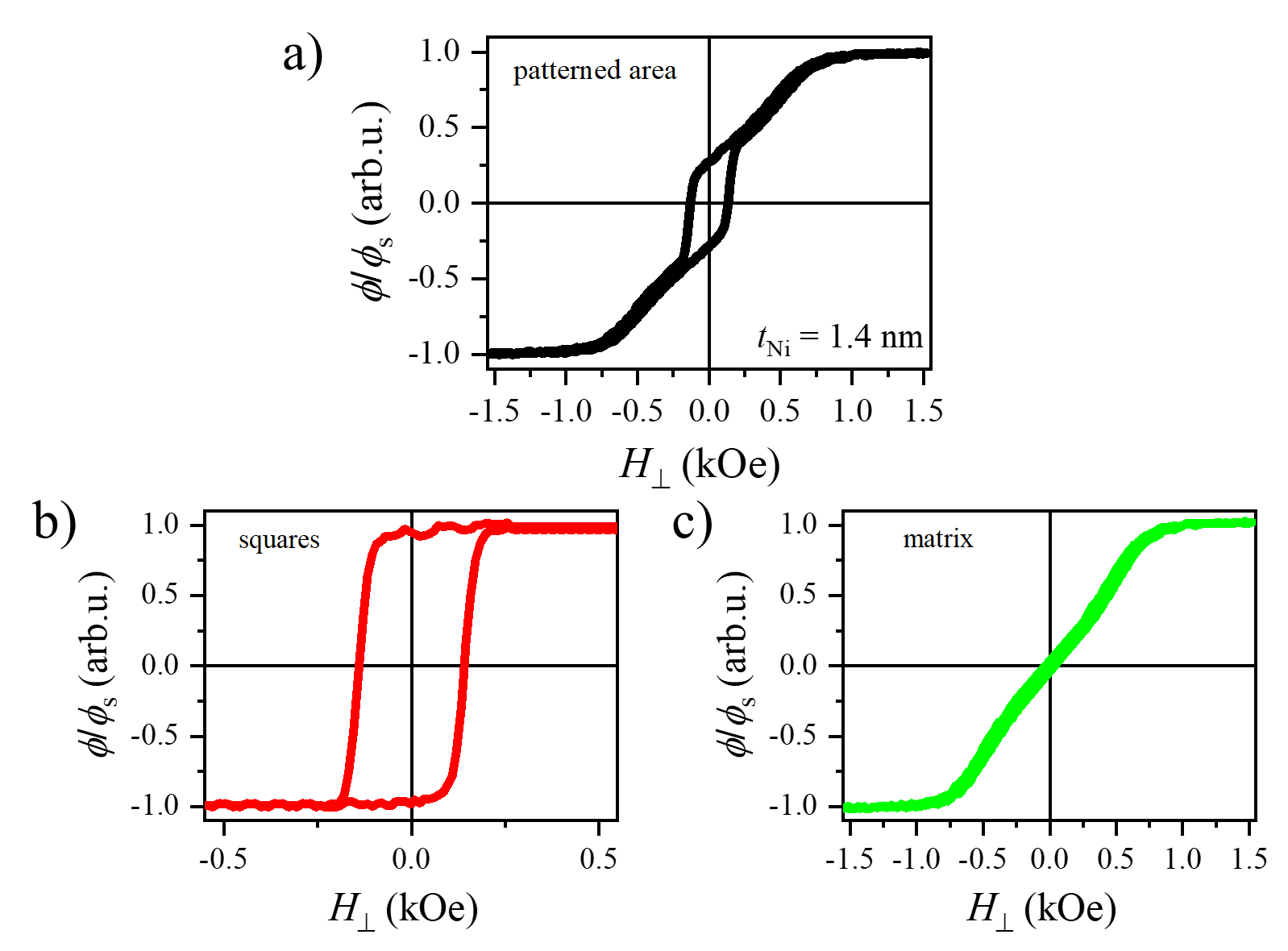


Figure S2. PMOKE hysteresis loop for buffer/Co1.4nm/Ni1.4 nm measured for the patterned area a), constituent PMOKE curves related to the magnetization reversal of the squares b) and matrix c) extracted from a).
